# New Appliances and Things Medical

**Published:** 1905-10-07

**Authors:** 


### NEW APPLIANCES AND THINGS MEDICAL.

**[We shall be glad to receive at our Office, 28 & 29 Southampton Street, Strand, London, W.C., from the manufacturers, specimens of all new preparations and appliances which may be brought out from time to time.]**

### SULPHAQUA BATH AND TOILET CHARGES, SULPHAQUA MEDICATED SOAP, SULOLA DENTIFRICE.

(The Seltzogene Patent Charges Co., St. Helens, Lancashire, England.)

The bath and toilet charges which we have received from the above source provide an exceedingly ingenious, efficacious, and convenient method of administering nascent sulphur baths. The efficacy of natural sulphur baths in certain forms of skin disease has been known from classical times. One of the main factors in rendering natural sulphur baths so much more active than artificial ones is the fact that in the former the sulphur is in a nascent condition. The charges which we have before us ensure the liberation in the bath or the washing basin of nascent sulphur. The charges consist of two powders which are to be separately dissolved in hot water, then mixed and thrown into the quantity of water requisite for the bath or toilet. When the directions are followed the resulting mixture becomes opalescent and gives off a distinct odour of sulphurous acid. The bath solution thus formed is exceedingly pleasant and invigorating, it contains no $H_2S$ and is thus free from all those objectionable properties which this substance imparts. We cannot doubt that these charges will be very popular and make much more generally available than heretofore the undoubtedly valuable therapeutic uses of sulphur when externally applied. We have also received a sample of sulphaqua medicated soap, which is certainly one of the best sulphur soaps of which we have any knowledge. The sulola dentifrice is also pleasant and possesses, according to the accompanying literature, pronounced antiseptic properties.

### THE ALLENBURY'S MILK AND CEREAL DIET, PANCREATISED.

(Allen and Hanburys, Ltd., London.)

The food before us consists of a fine white powder with a pleasant "biscuity" taste. It is made from pure milk rich in cream, and wholewheat, both these ingredients being predigested by the action of a pancreatic ferment during manufacture. The method of employment is to take one tablespoonful (heaped up) and add gradually to it one teacupful of boiling water. The resulting liquid can be again gently boiled for a minute or two, which will occasion slight thickening, or can be taken without boiling. If desired, milk can be used instead of water, but is unnecessary. The beverage when made according to the above directions is an exceedingly pleasant, refreshing and highly nutritious drink. We have found it well borne and very sustaining in acute and subacute diseases of the gastro-intestinal tract, and consider that it forms a valuable addition to the foods at present available in such conditions.

